# Supplementary material for: Quality Markers’ Discovery and Quality Evaluation of Jigucao Capsule Using UPLC-MS/MS Method
Source: Molecules. 2023 Mar 8;28(6):2494. doi: 10.3390/molecules28062494 (PMC10058756; doi:10.3390/molecules28062494)
Supplement: Supplementary file 1 [file molecules-28-02494-s001.zip › molecules-2234080-supplementary.pdf]

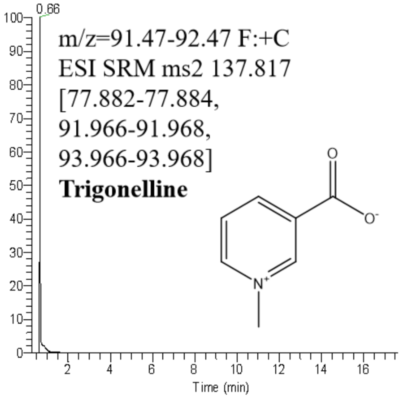
0.66
m/z=91.47-92.47 F:+C
ESI SRM ms2 137.817
[77.882-77.884,
91.966-91.968,
93.966-93.968]
Trigonelline
Time (min)

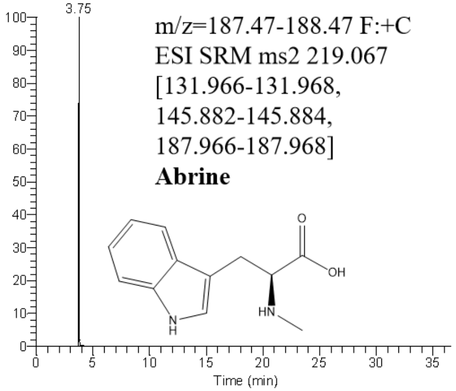
3.75
m/z=187.47-188.47 F:+C
ESI SRM ms2 219.067
[131.966-131.968,
145.882-145.884,
187.966-187.968]
Abrine
Time (min)

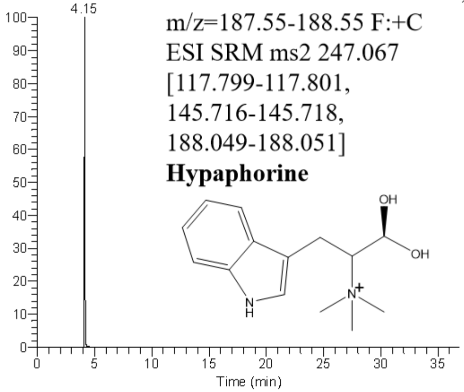
4.15
m/z=187.55-188.55 F:+C
ESI SRM ms2 247.067
[117.799-117.801,
145.716-145.718,
188.049-188.051]
Hypaphorine
Time (min)

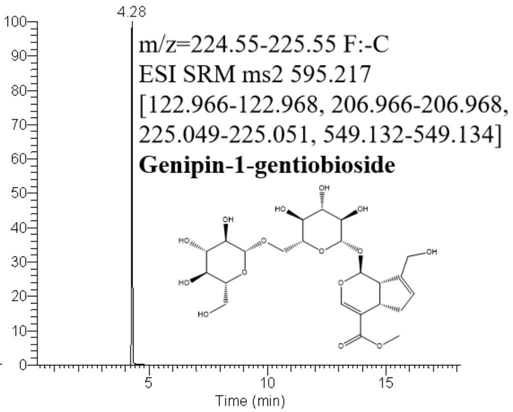
4.28
m/z=224.55-225.55 F:-C
ESI SRM ms2 595.217
[122.966-122.968, 206.966-206.968,
225.049-225.051, 549.132-549.134]
Genipin-1-gentiobioside
Time (min)

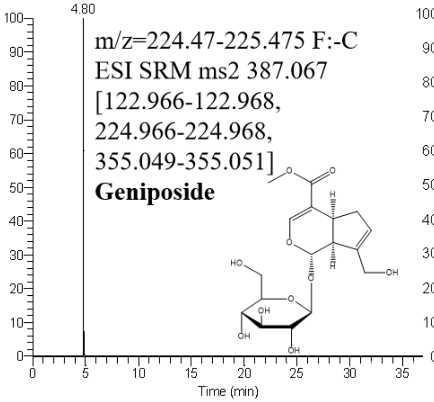
4.80
m/z=224.47-225.475 F:-C
ESI SRM ms2 387.067
[122.966-122.968,
224.966-224.968,
355.049-355.051]
Geniposide
Time (min)

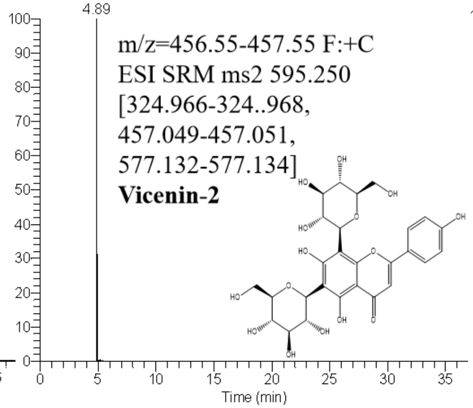
4.89
m/z=456.55-457.55 F:+C
ESI SRM ms2 595.250
[324.966-324..968,
457.049-457.051,
577.132-577.134]
Vicenin-2
Time (min)

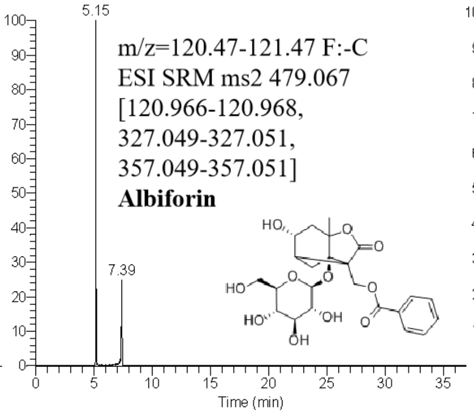
5.15
7.39
m/z=120.47-121.47 F:-C
ESI SRM ms2 479.067
[120.966-120.968,
327.049-327.051,
357.049-357.051]
Albiforin
Time (min)

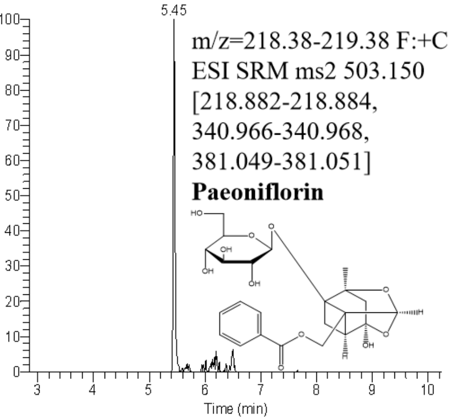
5.45
m/z=218.38-219.38 F:+C
ESI SRM ms2 503.150
[218.882-218.884,
340.966-340.968,
381.049-381.051]
Paeoniflorin
Time (min)

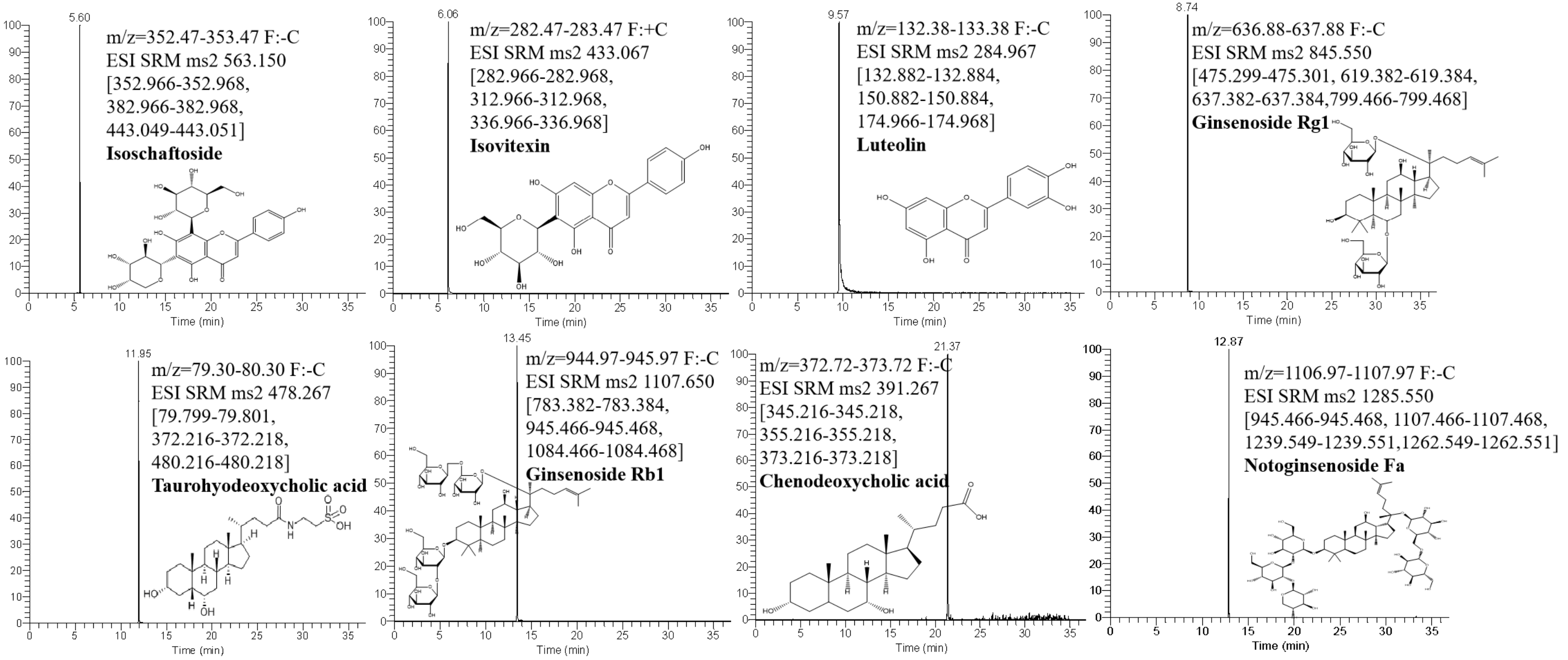

Figure S1. Mass spectrometric quantitative SRM chromatogram of compounds 1-16. The retention time, ion mode, structural formula and fragment ion information for each compound are included.

Table S1. The result of accuracy experimental

Compound	No	Measured amount(mg)	Added amount(mg)	Contained amount(mg)	Recovery rate(%)	Average recovery rate(%)	RSD (%)
	1	0.47	0.30	0.18	95.20		
	2	0.46	0.30	0.18	91.92		
	3	0.44	0.30	0.18	84.54		
	4	0.36	0.15	0.21	99.79		
Trigonelline	5	0.36	0.15	0.21	96.16	95.35	7.47
	6	0.36	0.15	0.22	91.87		
	7	0.26	0.08	0.19	98.89		
	8	0.27	0.08	0.20	90.12		
	9	0.27	0.08	0.18	109.65		
	1	0.96	0.60	0.31	108.16		
	2	0.95	0.60	0.31	107.06		
	3	0.94	0.60	0.31	104.87		
	4	0.65	0.40	0.30	88.63		
Abrine	5	0.65	0.40	0.30	89.07	97.92	8.06
	6	0.66	0.40	0.31	89.43		
	7	0.48	0.20	0.30	94.02		
	8	0.49	0.20	0.29	99.44		
	9	0.49	0.20	0.29	100.63		
	1	0.13	0.09	0.03	102.67		
	2	0.13	0.09	0.03	102.13		
	3	0.13	0.09	0.03	102.36		

	4	0.09	0.06	0.03	91.30		
Hypaphorine	5	0.09	0.06	0.03	93.07	94.42	8.77
	6	0.09	0.06	0.03	89.48		
	7	0.06	0.03	0.03	82.83		
	8	0.06	0.03	0.03	83.38		
	9	0.06	0.03	0.03	102.57		
	1	0.57	0.31	0.28	94.05		
	2	0.60	0.31	0.28	104.86		
	3	0.56	0.31	0.28	92.12		
	4	0.44	0.20	0.27	84.34		
Genipin-1-gentiobioside	5	0.46	0.20	0.27	92.85	95.04	8.26
	6	0.45	0.20	0.28	87.74		
	7	0.37	0.10	0.27	105.09		
	8	0.36	0.10	0.26	104.37		
	9	0.36	0.10	0.26	89.96		
	1	1.08	0.63	0.47	96.82		
	2	1.13	0.63	0.47	106.22		
	3	1.15	0.63	0.47	109.36		
	4	0.99	0.42	0.55	105.10		
Geniposide	5	0.98	0.42	0.55	103.84	103.73	7.37
	6	0.98	0.42	0.56	101.22		
	7	0.71	0.21	0.49	104.80		
	8	0.71	0.21	0.53	89.44		
	9	0.72	0.21	0.48	116.72		
	1	0.48	0.32	0.19	90.31		
	2	0.48	0.32	0.18	91.52		

	3	0.47	0.32	0.18	89.23		
	4	0.37	0.22	0.18	89.51		
Vicenin-2	5	0.38	0.22	0.18	95.38	93.21	7.89
	6	0.35	0.22	0.18	79.35		
	7	0.29	0.11	0.18	102.54		
	8	0.28	0.11	0.17	100.87		
	9	0.28	0.11	0.17	100.23		
	1	0.47	0.31	0.16	102.68		
	2	0.47	0.31	0.16	102.60		
	3	0.45	0.31	0.16	96.65		
	4	0.36	0.21	0.15	101.29		
Albiforin	5	0.37	0.21	0.15	107.22	98.62	6.51
	6	0.34	0.21	0.15	91.37		
	7	0.25	0.10	0.15	97.86		
	8	0.23	0.10	0.14	86.36		
	9	0.25	0.10	0.14	101.55		
	1	0.50	0.26	0.28	85.47		
	2	0.46	0.26	0.22	91.48		
	3	0.49	0.26	0.22	101.32		
	4	0.42	0.17	0.26	89.82		
Paeoniflorin	5	0.40	0.17	0.24	94.66	93.15	4.75
	6	0.38	0.17	0.22	92.02		
	7	0.34	0.09	0.26	95.01		
	8	0.31	0.09	0.23	96.18		
	9	0.33	0.09	0.25	92.34		
	1	0.92	0.49	0.43	97.94		

	2	0.94	0.49	0.43	103.54		
	3	0.91	0.49	0.43	97.71		
	4	0.71	0.33	0.42	88.79		
Isoschaftoside	5	0.73	0.33	0.42	95.49	95.51	7.88
	6	0.72	0.33	0.43	88.09		
	7	0.50	0.16	0.33	100.83		
	8	0.46	0.16	0.33	82.60		
	9	0.48	0.16	0.31	104.61		
	1	0.03	0.02	0.01	113.44		
	2	0.03	0.02	0.01	111.34		
	3	0.03	0.02	0.01	113.88		
	4	0.02	0.01	0.01	103.18		
Isovitexin	5	0.02	0.01	0.01	102.77	104.05	9.02
	6	0.02	0.01	0.01	107.17		
	7	0.01	0.01	0.01	106.46		
	8	0.01	0.01	0.01	90.22		
	9	0.01	0.01	0.01	88.01		
	1	0.42	0.25	0.16	105.62		
	2	0.41	0.25	0.16	102.66		
	3	0.39	0.25	0.16	94.68		
	4	0.34	0.16	0.16	110.72		
Ginsenoside Rg_1	5	0.33	0.16	0.16	106.33	107.96	9.48
	6	0.31	0.16	0.16	93.16		
	7	0.25	0.08	0.15	119.78		
	8	0.25	0.08	0.15	119.53		
	9	0.25	0.08	0.15	119.17		

Luteolin	1	0.03	0.02	0.01	114.59	105.01	7.00
	2	0.03	0.02	0.01	113.53		
	3	0.03	0.02	0.01	115.05		
	4	0.02	0.01	0.01	98.50		
	5	0.02	0.01	0.01	101.95		
	6	0.02	0.01	0.01	102.04		
	7	0.02	0.01	0.01	102.47		
	8	0.01	0.01	0.01	95.79		
	9	0.01	0.01	0.01	101.14		
Taurohyodeoxycholic acid	1	0.12	0.07	0.05	111.46	103.28	6.34
	2	0.12	0.07	0.05	112.69		
	3	0.12	0.07	0.05	109.89		
	4	0.09	0.04	0.05	103.86		
	5	0.09	0.04	0.05	101.25		
	6	0.09	0.04	0.05	99.15		
	7	0.07	0.02	0.05	96.98		
	8	0.07	0.02	0.05	95.27		
	9	0.07	0.02	0.05	98.98		
Notoginsenoside Fa	1	0.03	0.02	0.01	105.36	108.92	9.37
	2	0.03	0.02	0.01	119.04		
	3	0.03	0.02	0.01	120.19		
	4	0.02	0.01	0.01	115.15		
	5	0.02	0.01	0.01	117.02		
	6	0.02	0.01	0.01	106.41		
	7	0.01	0.01	0.01	88.28		
	8	0.01	0.01	0.01	107.03		

	9	0.01	0.01	0.01	101.82		
	1	0.42	0.33	0.13	88.32		
	2	0.41	0.33	0.13	86.61		
	3	0.40	0.33	0.13	82.28		
	4	0.34	0.22	0.12	99.10		
Ginsenoside Rb_1	5	0.34	0.22	0.12	98.49	94.90	9.34
	6	0.32	0.22	0.12	88.38		
	7	0.25	0.11	0.13	107.62		
	8	0.26	0.11	0.14	105.57		
	9	0.22	0.11	0.12	97.72		
	1	0.31	0.19	0.10	108.38		
	2	0.30	0.19	0.10	106.07		
	3	0.31	0.19	0.10	107.55		
	4	0.22	0.13	0.10	92.00		
Chenodeoxycholic acid	5	0.22	0.13	0.10	94.06	98.93	8.31
	6	0.24	0.13	0.10	108.06		
	7	0.16	0.06	0.10	91.04		
	8	0.15	0.06	0.10	90.67		
	9	0.15	0.06	0.10	92.57		

Table S2. The precision and stability results of 16 components in JGCC.

Number	Compounds	Repeability（mg/g）		Inter-day precision（mg/g）		Sample stability（mg/g）		Standard substance stability（peak area）	
		$\overline{X}$ ± SD	RSD (%)	$\overline{X}$ ± SD	RSD (%)	$\overline{X}$ ± SD	RSD (%)	$\overline{X}$ ± SD	RSD (%)
1	Trigonelline	1.96±0.07	3.76	1.93±0.12	6.16	1.93± 0.18	9.23	3,060,772± 45,446	1.48
2	Abrine	2.79±0.14	4.89	2.75±0.11	4.06	2.71± 0.20	7.50	51,665± 3557	6.89
3	Hypaphorine	0.30±0.01	3.30	0.30±0.01	3.79	0.32± 0.03	8.16	10,659,128± 826,788	7.76
4	Genipin-1-gentiobioside	2.50±0.03	1.25	2.51±0.06	2.49	2.50± 0.06	2.32	422,123±10,796	2.56
5	Geniposide	5.12±0.21	4.05	4.98±0.33	6.64	5.05± 0.18	3.63	22,618± 1109	4.90
6	Vicenin-2	1.66±0.04	2.55	1.64±0.06	3.65	1.66± 0.06	3.72	157,701± 6784	4.30
7	Albiforin	1.40±0.07	5.00	1.44±0.12	8.62	1.43± 0.11	7.43	35,224± 3018	8.57
8	Paeoniflorin	2.47±0.12	4.67	2.31±0.21	9.03	2.39± 0.23	9.48	1843± 150	8.13
9	Isoschaftoside	3.88±0.34	8.82	4.18±0.37	8.87	4.51± 0.25	5.53	330,539± 19,254	5.83
10	Isovitexin	0.07±0.00	2.13	0.07±0.00	3.38	0.07± 0.00	2.15	635,011± 16,019	2.52
11	Ginsenoside Rg_1	1.45±0.08	5.40	1.49±0.10	6.83	1.49± 0.12	8.19	182,968± 15,432	8.43
12	Luteolin	0.09±0.00	2.10	0.09±0.00	3.97	0.09± 0.00	2.62	955,423± 46,387	4.86
13	Taurohyodeoxycholic acid	0.45±0.03	7.76	0.42±0.03	7.78	0.43± 0.04	9.04	324,888± 19,584	6.03
14	Notoginsenoside Fa	0.06±0.00	7.71	0.06±0.00	8.89	0.05± 0.01	9.64	14,725± 1182	8.03
15	Ginsenoside Rb_1	1.13±0.09	8.19	1.16±0.10	8.86	1.15± 0.08	7.21	6187± 440	7.11
16	Chenodeoxycholic acid	0.93±0.07	7.75	0.87±0.08	9.23	0.85± 0.07	8.45	2753± 270	9.81

$\overline{X}$: average value; SD: standard deviation; RSD: relative standard deviation.

Table S3. The complete information of JGCC and *Zingiber officinale* Rosc.

Name	Batch numbers	Manufacturers
JGCC	2111095	Guangxi yulin pharmaceutical group Co., Ltd.
JGCC	2105033	
JGCC	2111092	
JGCC	2111093	
JGCC	2111094	
JGCC	2102006	
JGCC	2102007	
JGCC	2102008	
JGCC	2101005	
JGCC	2101003	
JGCC	2111091	
JGCC	2111099	
JGCC	2108070	
JGCC	2108069	
Zingiber officinale Rosc	20201201	Guangxi Xianzhu Chinese Medicine Technology Co., Ltd

Table S4. Series concentration of mixed standard solution for the 16 different active ingredients in JGCC (ng/mL).

Number	Compounds	Batch No	1	2	3	4	5
1	Trigonelline	wkq20052702	12.18	121.80	243.60	609.00	24,360.00
2	Abrine	B01A6L2047	9.98	99.80	499.00	19,960.00	99,800.00
3	Hypaphorine	wkq20070902	15.28	152.80	305.60	764.00	30,560.00
4	Genipin-1-gentiobioside	wkq20021909	10.22	102.20	511.00	20,440.00	102,200.00
5	Geniposide	wkq18010506	13.89	138.90	694.50	27,780.00	138,900.00
6	Vicenin-2	wkq20030503	7.21	72.10	360.50	14,420.00	72,100.00
7	Albiforin	wkq18052205	10.31	103.10	515.50	20,620.00	103,100.00
8	Paeoniflorin	wkq18032104	115.10	575.50	2877.50	23,020.00	115,100.00
9	Isoschaftoside	wkq20061812	10.93	109.30	546.50	21,860.00	109,300.00
10	Isovitexin	Y13D9H77613	10.76	107.60	215.20	538.00	2690.00
11	Ginsenoside Rg_1	wkq18050401	10.93	109.30	218.60	546.50	21,860.00
12	Luteolin	wkq19031213	11.16	111.60	223.20	228.00	2790.00
13	Taurohyodeoxycholic acid	B16A10K94146	10.98	109.80	219.60	549.00	21,960.00
14	Notoginsenoside Fa	wkq18030709	105.10	210.20	525.50	2627.5	21,020.00
15	Ginsenoside Rb_1	wkq18020808	11.02	110.20	220.40	551.00	22,040.00
16	Chenodeoxycholic acid	wkq20051511	10.50	105.00	210.00	525.00	21,000.00

Table S5. Retention time (t_R), optimized parent ion (MS_1), fragment ion (MS_2), collision energy (CE), radio frequency voltage (RF) of 16 active ingredients.

Number	Compounds	t_R（min）	Ion form	MS_1	MS_2	CE（V）	RF （V）
1	Trigonelline	0.66	$[M+H]^+$	137.82	91.97	22	105
2	Abrine	3.75	$[M+H]^+$	219.07	187.97	12	84
3	Hypaphorine	4.15	$[M+H]^+$	247.07	188.05	13	80
4	Genipin-1-gentiobioside	4.29	$[M-H]^-$	549.13	225.05	19	143
5	Geniposide	4.80	$[M-H]^-$	387.07	224.97	8	174
6	Vicenin-2	4.89	$[M+H]^+$	595.25	457.05	19	179
7	Albiforin	5.15	$[M-H]^-$	479.07	120.97	19	202
8	Paeoniflorin	5.43	$[M+Na]^+$	503.15	218.88	27	299
9	Isoschaftoside	5.60	$[M-H]^-$	563.15	352.97	35	299
10	Isovitexin	6.06	$[M+H]^+$	433.07	282.97	26	144
11	Ginsenoside Rg_1	8.74	$[M-H]^-$	799.47	637.38	30	195
12	Luteolin	9.57	$[M-H]^-$	284.97	132.88	34	193
13	Taurohyodeoxycholic acid	11.96	$[M-H]^-$	498.27	79.80	68	299
14	Notoginsenoside Fa	12.87	$[M-H]^-$	1239.55	1107.47	55	165
15	Ginsenoside Rb_1	13.46	$[M-H]^-$	1107.65	945.47	42	299
16	Chenodeoxycholic acid	21.37	$[M-H]^-$	391.27	373.22	32	299
